# Association of Mesiodentes and Dens Invaginatus in a Child: A Rare Entity

**DOI:** 10.1155/2012/198032

**Published:** 2012-11-06

**Authors:** A. N. Sulabha, C. Sameer

**Affiliations:** ^1^Department of Oral Medicine and Radiology, Al-Ameen Dental College and Hospital, Athani Road, Karnataka, Bijapur 586108, India; ^2^Department of Oral and Maxillofacial Surgery, Al-Ameen Dental College and Hospital, Karnataka, Bijapur 586108, India

## Abstract

Supernumerary teeth are defined as any teeth in excess of normal number. Mesiodens is a supernumerary tooth, in the central region of premaxilla between two central incisors. Dens invaginatus is a developmental anomaly resulting from invagination in the surface of tooth crown before calcification has occurred. Radiographically, it is observed as infolding of a radioopaque ribbon like structure, with equal density as enamel, extending from cingulum into a root canal and sometimes reaching the root apex. This paper aims to present a rare association of dens invaginatus with two mesiodentes in a child causing the eruption disturbance and unaesthetic appearance in anterior maxilla.

## 1. Introduction

Supernumerary teeth or hyperdontia is defined as excess number of teeth as compared to the normal dental formula [1]. The most common supernumerary tooth as indicated by Alberti is mesiodens. A mesiodens is a supernumerary tooth located in maxillary central incisor region. Mesiodens may occur as single, multiple, unilateral, or bilateral. Multiple mesiodens are called mesiodentes [2, 3]. Single supernumerary teeth account for 76–86%, in pair accounts for 12–23% and less than 1% cases with three or more extra teeth [4].

Dens invaginatus is a rare malformation of teeth showing a broad spectrum of morphological variation [5]. It is a developmental anomaly resulting from invagination of enamel organ into a dental papilla, beginning at the crown and sometimes extending into the root before calcification occurs [6]. Although a clinical examination reveals a deep pit or fissures on lingual surfaces of anterior teeth, the radiographic examination is the sine quo non for diagnosis of dens invaginatus [7].

 Association of dens invaginatus with mesiodens is a very rare phenomenon. Extensive Pubmed search revealed only five case reports published in literature till date [8–10]. This paper aims to report a rare association of dens invaginatus in two unusual mesiodens in a child causing the eruption failure of permanent teeth and unaesthetic appearance.

## 2. Case Report

**Figure 1:**
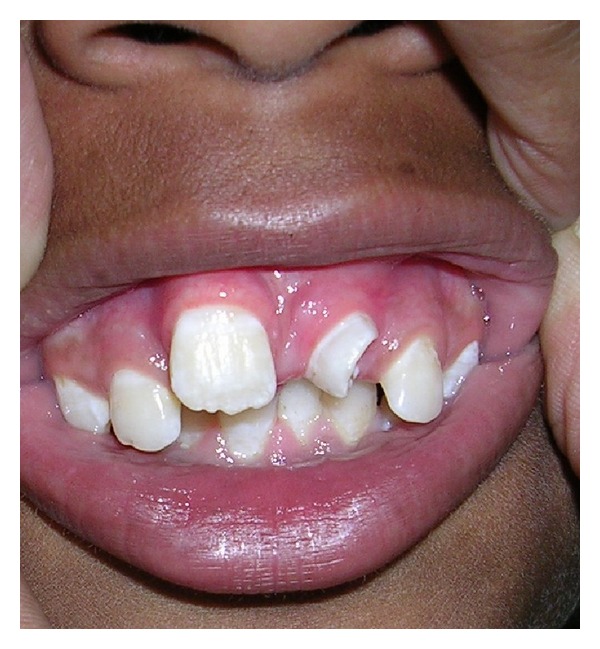
Clinical picture showing horizontal partial eruption on mesiodens.

**Figure 2:**
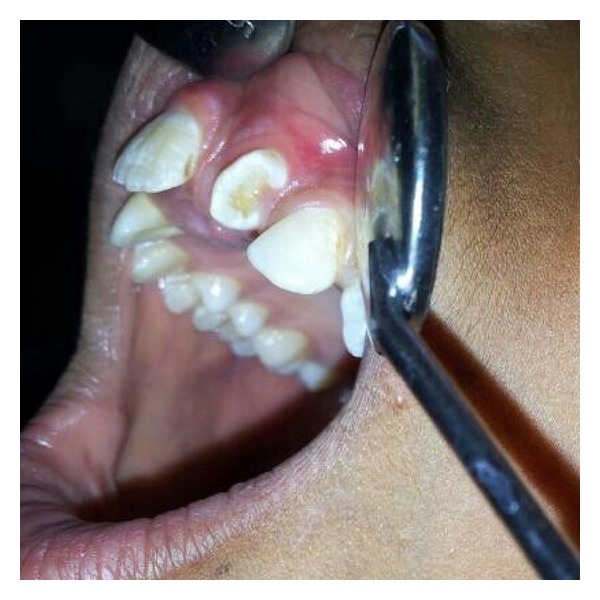
Clinical picture showing the lateral aspects of mesiodens.

A 13-years-old child reported with complaint of abnormally erupted tooth in maxillary anterior region (Figure 1). Intraoral examination revealed partial horizontally erupted tooth in left central incisor region. Only incisal and part of middle one third of the abnormally erupted tooth was visible. The lingual surface appeared abnormal with infolding of mesial and distal edges towards centre creating a central depressed area (Figure 2). 

**Figure 3:**
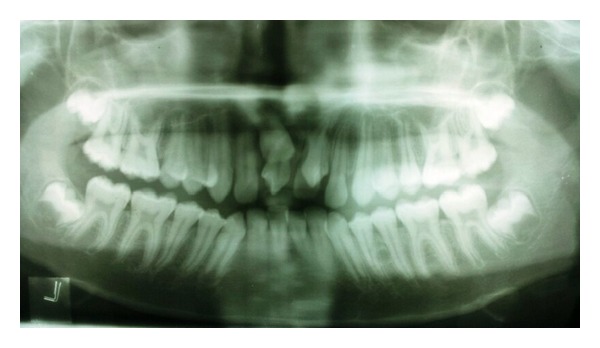
Panoramic view showing the two mesiodens in the anterior region of maxilla with unerupted left central incisor.

Radiographically examination revealed unerupted left central incisor. Two supernumerary teeth were found in maxillary central incisor region. One of the supernumerary teeth was partially erupted in left central incisor region in horizontal manner and the other was impacted. Root appeared to be incomplete (Figure 3). Partial erupted mesiodens showed invagination of radioopaque line towards the pulp suggesting dens invaginatus. As these mesiodens caused eruption failure of left central incisor and was esthetically unpleasant, extraction of both mesiodens was done. After extraction, patient was advised on wait and watch policy for eruption of the left central incisor.

**Figure 4:**
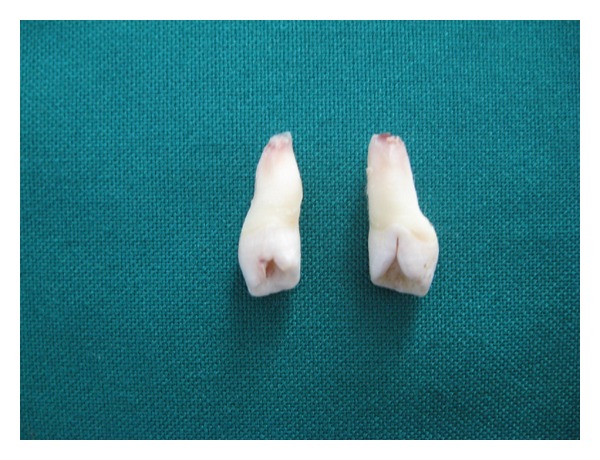
Lingual aspect of both mesiodens showing the dens invaginatus.

**Figure 5:**
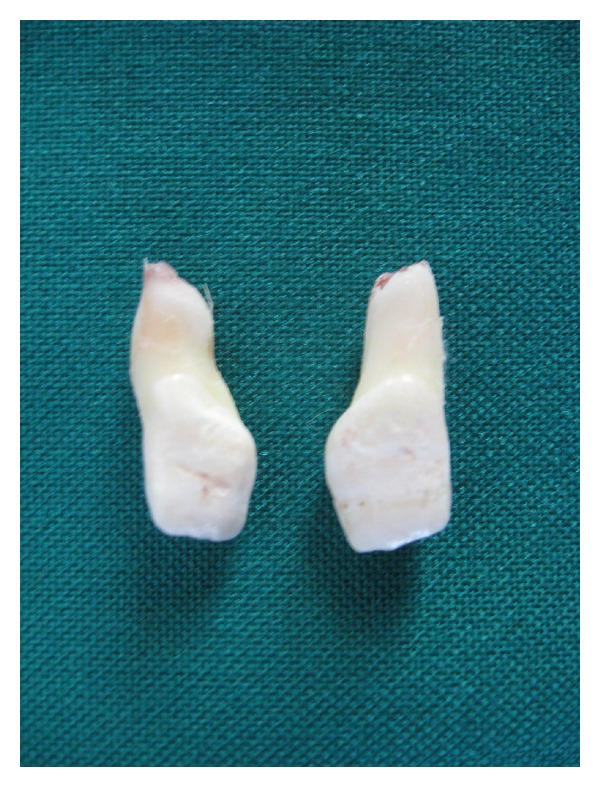
Labial aspect of mesiodens showing the dilaceration and indentation on crown of mesiodens.

**Figure 6:**
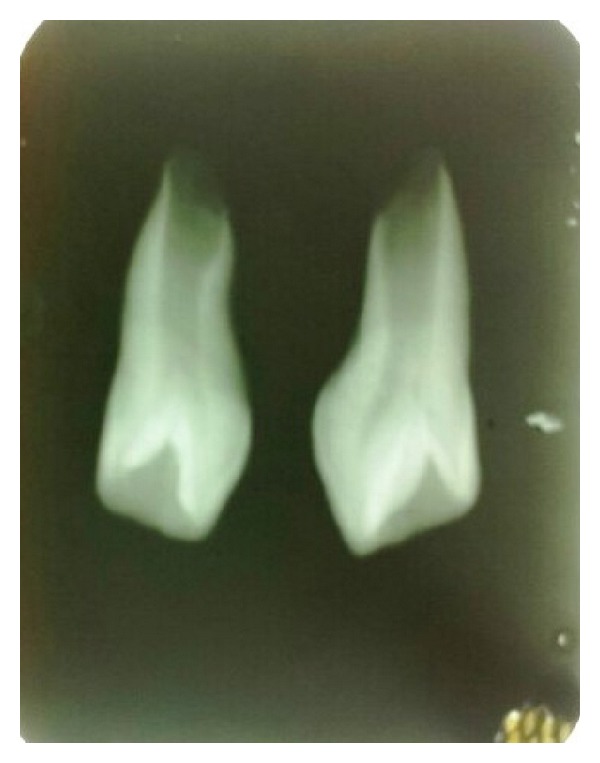
Postoperative X-ray showing incomplete root formation of mesiodens.

Extracted mesiodentes were unusual (Figures 4 and 5). Partially erupted mesiodens was bigger with crown morphology resembling central incisor, other had smaller crown morphology resembling the lateral incisor. Root formation was incomplete with wide open apex with both mesiodentes. The labial surface showed some indentation. The lingual surface of both showed complete infolding of both mesial and distal edges till midline giving a central depressed area and extended till cervical part of root, bifurcating the pulp without invading it (Figure 6). Both mesiodentes showed dilacerations, smaller mesiodens in root portion, and bigger in the crown portion. Based on these a diagnosis of mesiodens with dens invaginatus and dilaceration was made.

## 3. Discussion

Supernumerary teeth are developmental disturbances occurring during the odontogenesis resulting in the formation of teeth in excess of the normal number. Mesiodens refers to supernumerary tooth in the premaxilla between the two central incisor and these are more common in the permanent dentition than in primary dentition [11].

Mesiodentes can be classified on basis of their occurrence in permanent dentition (rudimentary) and according to their morphology as conical, tuberculate, molariform, or supplemental. Most commonly mesiodens presents in conical shape. Tuberculate mesiodentes are barrel shaped with several cusp or tubercles and have incomplete roots or abnormal root formation. They rarely erupt into the oral cavity. The rare form is molariform mesiodens. Supplemental mesiodens resembles natural teeth in both size and shapes are usually seen at end of tooth series. Supplemental maxillary incisors are much less common than conical or tuberculate supernumerary teeth in an anterior maxilla. Supplementary lateral incisor is more common than supplemental central incisor [12, 13]. In the present case the crown resembled supplemental central and lateral incisor but had incomplete root. Both mesiodentes showed the dilaceration with dens invaginatus.

Dens invaginatus is a developmental malformation resulting from invagination of the tooth crown or root before calcification has occurred [6]. The etiology of this is unknown and controversial. In most cases it is detected by chance on radiograph. Clinically an unusual crown (dilated, peg shaped, barrel shaped) or deep foramen coecum may be an important hint [5]. Radiographically it is observed as infolding of a radioopaque ribbon like structure with equal density as enamel extending from cingulum into root canal and sometimes reaching the root apex, assigning the appearance of a small tooth within the coronal pulp cavity [8].

Oehlers classification is most commonly used for the dens invaginatus [5].

 Type 1: an enamel lined minor form occurs in the crown of the tooth and not extending beyond the cemento enamel junction.
 Type 2: an enamel lined form which invades the root but remains confined as blind sac. It may or may not communicate with dental pulp.
 Type 3: a form which penetrates through the root perforating at the apical area showing a second foramen in the apical or in the periodontal area. There is no immediate communication with the pulp. In the present case both mesiodentes had a blind sac extending to pulp and dividing it without communicating.

This anomaly occurs frequently in lateral incisors followed by central incisor, premolars canines, and molars [7]. Association of this anomaly with the mesiodens is extremely rare and its occurrence in two mesiodentes is even a rarer phenomenon. Review of English language literature only showed five case reports of dens invaginatus in mesiodens and among them involvement of two mesiodentes is limited to only two case reports [8–10]. Sannomiya et al. [8] presented two cases of mesiodens and dens invaginatus of which one case presented with two mesiodentes associated with dens invaginatus. Archer and Silverman [10] presented dens invaginatus in bilateral rudimentary supernumerary teeth. In the present case dens invaginatus was noted with different types of mesiodens having supplemental crown morphology of central and lateral incisor with incomplete root and dilaceration which is very rare and unusual.

Various complications might occur as a result of the presence of supernumerary teeth and dens invaginatus. Delayed eruption, crowding, spacing, impaction, diastema, cystic lesion, root resorption, and so forth are complications associated with supernumerary teeth. The dens invaginatus in dens in dente allows entry of irritants into an area which is separated from pulpal tissue by only a thin layer of enamel and dentine and presents a predisposition for development of caries. Pulpal necrosis, abscess formation, cyst, and internal resorption are other complications [3, 5]. In the present case partial erupted mesiodens in horizontal manner gave unaesthetic appearance along with eruption failure of the left central incisor.

 Supernumerary teeth either can be managed by removal, endodontic treatment or can be monitored without its removal [13, 14]. In this case as it was associated with unaesthetic appearance and eruption failure of permanent teeth, surgical removal of both mesiodentes was done.

To conclude as mesiodentes are associated with various complications, early diagnosis and treatment are very important to prevent physiological, esthetics, and functional problems especially in children.
